# Adénome pléomorphe intra-sinusien maxillaire: une entité rare

**DOI:** 10.11604/pamj.2015.22.81.7924

**Published:** 2015-10-01

**Authors:** Rex Mario Razafindrakoto, Mahamad Rojovolaarivony Schammirah

**Affiliations:** 1 Service d'Oto-rhino-laryngologie et de Chirurgie Cervico-faciale, Centre Hospitalier Universitaire d'Andohatapenaka, Antananarivo, Madagascar

**Keywords:** adénome pléomorphe, chirurgie, sinus maxillaire, tomodensitométrie, pleomorphic adenoma, surgery, maxillary sinus, CT scan

## Image en medicine

Les adénomes pléomorphes, retrouvés le plus souvent au niveau des glandes salivaires, sont localisés exceptionnellement dans un sinus de la face. Notre observation concerne un patient de genre masculin, âgé de 17 ans, qui a présenté depuis un an, du côté gauche, une obstruction nasale, une rhinorrhée muqueuse, une épistaxis, une douleur infra-orbitaire irradiant vers les dents maxillaires et un larmoiement. L'inspection a montré une hémiface fortement déformée par une tuméfaction lisse faisant huit centimètres de grand axe (A,B), de consistance dure à la palpation. La rhinoscopie antérieure a révélé dans la cavité nasale gauche une tumeur lisse, blanchâtre, non hémorragique, non infectée, refoulant le septum. L'examen des paires des nerfs crâniens a été sans particularités. Un examen du fond d’œil et du champ visuel a été dans les limites de la normale. Les aires ganglionnaires cervicales ont été libres. Un examen tomodensitométrique du massif facial, avec et sans injection de produit de contraste, en reconstruction tridimensionnelle (C), en coupes coronales, axiales et sagittales (D,E,F), a objectivé une masse refoulant sans les lyser les structures osseuses et cartilagineuses avoisinantes (toit de l'ethmoïde, parois orbitaires, voûte palatine, septum nasal). Un prélèvement tumoral suivi d'un examen anatomopathologique a confirmé le diagnostic d'adénome pléomorphe. Une chirurgie d'exérèse a été menée par voie paralatéronasale (G), permettant d'enlever la tumeur en monobloc (H). Les suites opératoires ont été bonnes, les signes fonctionnels se sont progressivement amendés. Il n'y a pas eu de récidive tumorale six mois après l'intervention.

**Figure 1:**
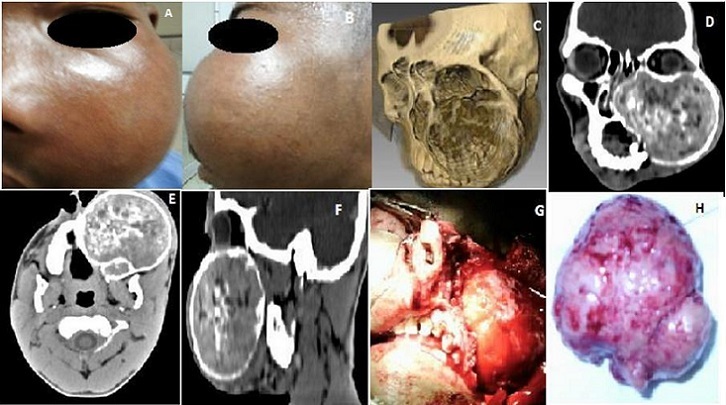
(A): photographie du patient porteur de l'adénome pléomorphe intra-sinusien maxillaire gauche, vue de face; (B): photographie du patient porteur de l'adénome pléomorphe intra-sinusien maxillaire gauche, vue de profil; (C): cliché tomodensitométrique en reconstitution tridimensionnelle; (D): cliché tomodensitométrique en coupe coronale et avec injection de produit de contraste; (E): cliché tomodensitométrique en coupe axiale et avec injection de produit de contraste; (F): cliché tomodensitométrique en coupe sagittale et avec injection de produit de contraste; (G): vue per-opératoire de l'ablation de l'adénome pléomorphe intra-sinusien maxillaire par voie paralatéronasale; (H): pièce opératoire

